# Obstruction of Bowels in a Horse, Due to Abscess in the Lumbosacral Region1Read before the Fifteenth Annual Meeting of the Iowa State Veterinary Medical Association, Cedar Rapids, Iowa, January 14 and 15, 1903.

**Published:** 1903-02

**Authors:** S. H. Bauman

**Affiliations:** Birmingham, Iowa


### OBSTRUCTION OF BOWELS IN A HORSE, DUE TO ABSCESS IN THE LUMBOSACRAL REGION.[1]

By S. H. Bauman, D.V.S.,
BIRMINGHAM, IOWA.

In 1898 I was called to a case of colic, but the horse died before I arrived. The history of the case was that the horse had shown a few colicky symptoms for more than a week, but, as he was in pasture and kept eating, the owner paid little attention to him. But on the day I was called he found the horse in a serious condition, with result as stated above.

I found, in making the post-mortem examination, all organs normal except the bowels, which were impacted, and especially so was the floating colon. After removing the bowels, and not being satisfied that I had found a sufficient cause for death, I continued my examination and found an abscess immediately beneath the lumbosacral articulation. The abscess was almost as large as the crown of a hat, and had pressed up on the rectum so as to completely obstruct the bowels. On opening the abscess, which was about ready to break, I found over a pint of pus. Since that time I have had quite a number of these cases, and whenever I have an obstinate case of impaction, especially where the horse points high, I make an examination, and it is surprising the number I have found. All these abscesses are similar and in the same location. The last one I had was December 26, 1902, in a fine mare. A description of this case will answer for all. The horse is drowsy, paws a little once in a while, lies stretched flat on the ground, turns the head to the side and points very high, as a rule—but I have had them where

[1] Read before the Fifteenth Annual Meeting of the Iowa State Veterinary Medical Association, Cedar Rapids, Iowa, January 14 and 15, 1903.

they pointed low—gets up, grazes for a time, and lies down as before, and remains in this position for quite a while; have never seen any bloating in these cases. The only treatment required is to remove with the hand all the feces and repeat this as often as the back bowels become obstructed. When this is done the horse will resume eating. As soon as the abscess is sufficiently developed either break through the rectum with the finger or cut through with a short, hooked knife and evacuate the abscess. No medicine is required in any of these cases except carbolized oil. Do not allow the animal to have much bulky food. Many of these cases will break quickly, and the horse will recover of his own accord. All the cases I have had recovered, but I have had several which formed satellites, which required treatment as above for ten to fifteen days. There are no external symptoms as in cases of furunculus close to the anus, in which we always have external swelling. The only theory I can advance as to the cause of these abscesses would be some injury to the vertebræ at this point, or possibly a severe sprain.

